# Managing Choroidal Neovascularization in Pseudoxanthoma Elasticum: Outcomes of Vitrectomy and Intravitreal Ranibizumab/Aflibercept Therapy—A Case Report

**DOI:** 10.1155/crop/9286332

**Published:** 2025-05-21

**Authors:** Kazuya Yamashita, Kento Hashizume, Yasumasa Fukuda, Rio Sato, Nobuhiro Ozawa, Hirohisa Kubono, Mari Kawamura, Kotaro Suzuki

**Affiliations:** ^1^Department of Ophthalmology, Keiyu Hospital, Yokohama, Kanagawa, Japan; ^2^Department of Ophthalmology, National Hospital Organization Tokyo Medical Center, Meguro-ku, Tokyo, Japan; ^3^Kyorin Eye Center, Kyorin University School of Medicine, Mitaka, Tokyo, Japan

**Keywords:** aflibercept, angioid streaks, anti-VEGF therapy, choroidal neovascularization, intravitreal injections, pseudoxanthoma elasticum, ranibizumab, submacular hemorrhage, vitrectomy

## Abstract

**Background:** Pseudoxanthoma elasticum (PXE) is a systemic disorder that affects the skin, eyes, and vascular system. It commonly presents with retinal angioid streaks (ASs) and can lead to vision loss due to subretinal neovascularizations and macular atrophy. Diagnosis is confirmed through skin biopsies showing calcified elastic fibers or identifying biallelic ABCC6 pathogenic variants. This case report is novel as it describes the clinical course of choroidal neovascularization (CNV) secondary to PXE treated with vitrectomy and intravitreal ranibizumab and aflibercept injections in both eyes.

**Case Presentation:** A 68-year-old woman presented with vision loss in her right eye. Her medical history included hypertension, uterine fibroids, and multiple drug allergies. Ophthalmic examination revealed radial AS around the optic discs and subretinal hemorrhages in the right eye. Fluorescein angiography and optical coherence tomography confirmed CNV and fresh subretinal hemorrhage. A series of vitrectomies and intravitreal injections of ranibizumab and aflibercept were performed to manage the CNV and submacular hemorrhage. Despite recurrence, subsequent surgeries stabilized her condition, improving her best-corrected visual acuity to 20/125 in the right eye over 6 years. A skin biopsy confirmed the diagnosis of PXE, a condition she had overlooked for over 30 years.

**Conclusions:** This case emphasizes the importance of early detection of AS through thorough fundus examination, alongside comprehensive evaluation for systemic conditions. Management of CNV in PXE involves the prompt use of intravitreal anti-VEGF injections and vitrectomy with tissue plasminogen activator (tPA) for controlling CNV activity and submacular hemorrhage. Ophthalmologists should consider PXE in patients presenting with characteristic skin and eye findings and refer them for dermatological evaluation as necessary.

## 1. Background

Pseudoxanthoma elasticum (PXE) is a systemic disorder affecting the skin, eyes, and vascular system, often presenting with retinal angioid streaks (ASs) or skin papules. The primary cause of disability is vision loss due to subretinal neovascularizations and macular atrophy. Diagnosis is based on characteristic skin and eye findings, confirmed by identifying calcified elastic fibers in skin biopsies or biallelic ABCC6 pathogenic variants [1]. The main characteristic of fundus imagery examination is AS, which are fractures of Bruch's membrane (BM). In this case, we administered vitrectomy for submacular hemorrhage (SMH), several intravitreal injections with ranibizumab and aflibercept in the right eye, and several intravitreal injections with aflibercept in the left eye to address secondary choroidal neovascularization (CNV) of PXE. There have been many reports of PXE accompanied by pigmented streaks due to vascular endothelial growth factor inhibitors and photodynamic therapy [2–5]. To the best of our knowledge, this article may be the first to provide clinical course of CNV secondary to PXE treated by vitrectomy and intravitreal ranibizumab and aflibercept injections in each eye. Additionaly, the patient had noticed skin symptoms about 30 years before the onset of ophthalmic symptoms but had not visited a dermatologist. Recent eye symptoms prompted a skin lesion biopsy, leading to a diagnosis of PXE. At present, no vascular lesions have been observed in the head or heart. Ophthalmologists should pay attention to skin lesions when making a diagnosis.

## 2. Case Presentation

A 68-year-old woman was referred to the Ophthalmology Department of Keiyu Hospital on July 2, 2018, for vision loss in her right eye, which began the previous day. Her medical history includes high blood pressure and uterine fibroids. The patient did not have a known family history of PXE. Genetic testing was not performed due to limitations in access and cost at the time of diagnosis. She is allergic to contrast media used during CT scans, as well as the drugs Buscopan, penicillin, and indocyanine green (ICG). She has no history of cerebral or myocardial infarction. The patient recalled a retinal hemorrhage caused by a tennis ball hitting her eye about 10 years ago, but she could not remember which eye was affected. She had no history of eye surgery and no family history of similar eye symptoms.

**Figure 1:**
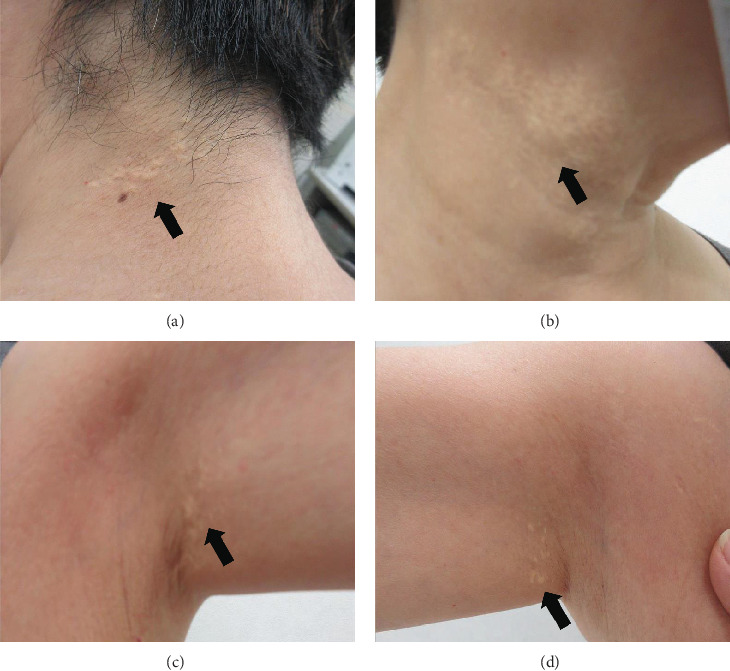
The black arrow represents yellow, soft, papular changes. (a) Left posterior neck. (b) Right anterior neck. (c) Left armpit. (d) Right armpit.

**Figure 2:**
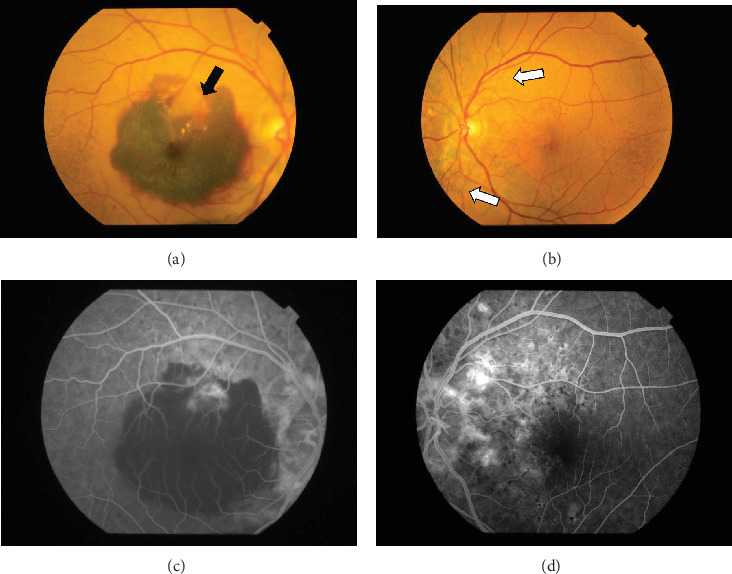
Fundus color photos taken on July 2, 2018. (a) Right eye. (b) Left eye. The retinal–choroidal atrophy shows around the optic disc in both eyes. In the right eye, the extensive dark-red hemorrhage was seen around the macula, and the black arrows indicate an orange-red lesion that was found above the macula. The white arrows indicate brownish-black AS radiating from the optic disc in the left eye. Fluorescein fundus angiography taken on July 2, 2018. (c) Right eye. (d) Left eye. In the late stage of bilateral angiography, presented radial hyperfluorescence around the optic disc represents AS. The hyperfluorescence represents the leakage area of CNV.

**Figure 3:**
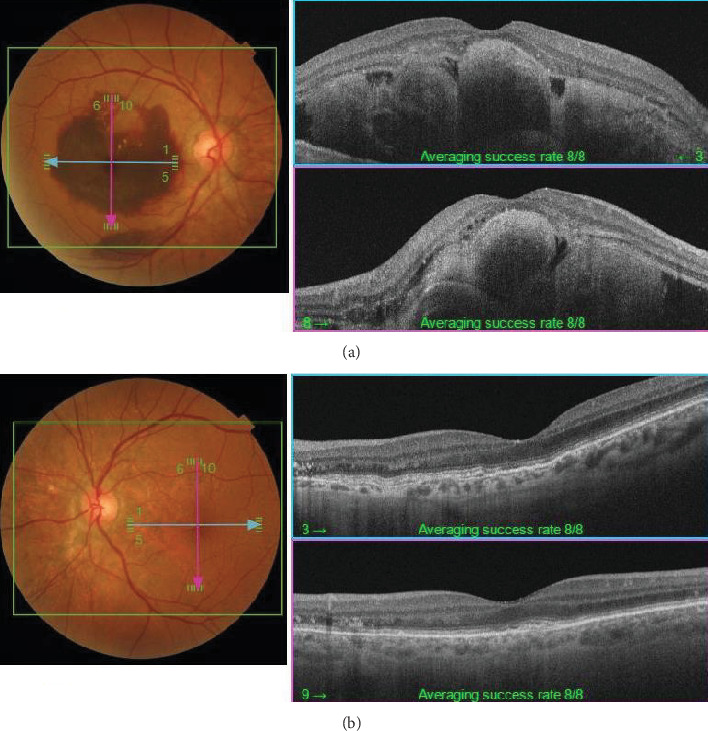
OCT taken on July 2, 2018. (a) Right eye. (b) Left eye. (a) Fresh, dense bleeding is observed under the retina in the macular area. (b) Irregular ellipsoid zones are observed around the optic nerve due to CNV derived from AS.

The patient had noticed skin symptoms on her neck, nape, and armpits since her 30s but had not sought medical attention. Examination revealed yellow, soft, papular changes on the anterior neck, nape, and both armpits (Figure 1). Her best corrected visual acuity (BCVA) was 20/200 in the right eye and 20/20 in the left eye. Ophthalmological examination findings included intraocular pressures of 17 mmHg in the right eye and 16 mmHg in the left eye, with no abnormalities in the anterior segments of either eye. The axial length was 22.50 mm in the right eye and 22.70 mm in the left eye (IOL Master 500, Carl Zeiss Meditec AG, Germany). Fundus examination revealed retinal–choroidal atrophy around the optic disc in both eyes, extensive dark-red hemorrhage was seen around the macula, and an orange-red lesion was found above the macula in the right eye (Figure 2a,b). Fluorescein angiography detected hyperfluorescence around the optic discs and areas of CNV leakage in both eyes which represent AS, and the right eye had subretinal hemorrhage with widespread hypofluorescence in the posterior pole (Figure 2c,d). Optical coherence tomography (OCT) confirmed extensive fresh subretinal hemorrhage and CNV in the posterior pole involving the macula in the right eye and retinal pigment epithelial irregularity suggestive of CNV in the left eye (Figure 3).

**Figure 4:**
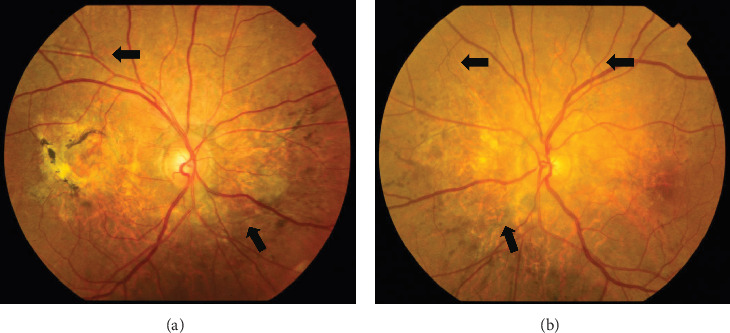
Fundus color photos taken after vitreous surgery on October 24, 2022. (a) Right eye. (b) Left eye. The “peau d'orange” appearance of the posterior fundus is centered around the optic disc in both eyes. The black arrows indicate brownish-black AS radiating from the optic disc.

**Figure 5:**
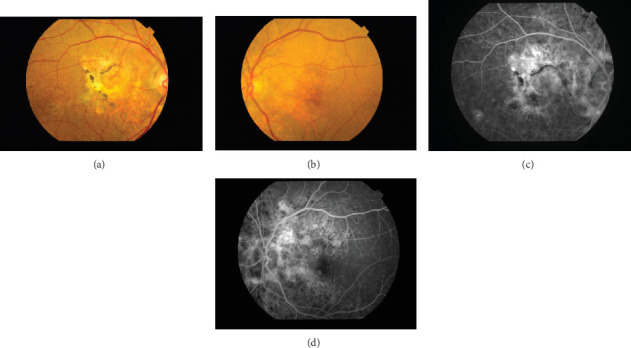
Fundus color photos taken after vitreous surgery on October 24, 2022. (a) Right eye. (b) Left eye. AS present radially around the optic discs of both eyes. The retina of the posterior pole area of the right eye shows that organized subretinal hematoma leaves scarring. The retina of the posterior pole area of the left eye shows that choroidal neovascularization can be found in near areas of the optic nerve and the macular area. Fluorescein fundus angiography taken after vitreous surgery on October 24, 2022. (c) Right eye. (d) Left eye. In the late stage of bilateral angiography, radial hyperfluorescence was presented around the optic disc—the hyperfluorescence corresponding to the AS, with the pigmented areas within the streaks showing hypofluorescence.

**Figure 6:**
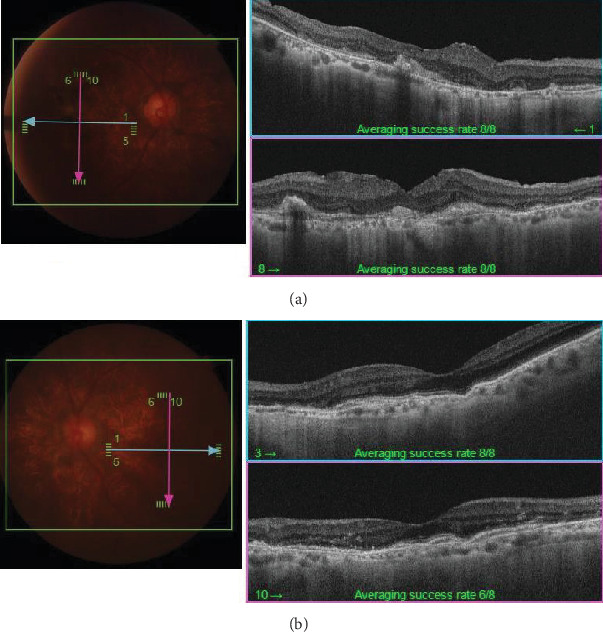
OCT taken on June 10, 2024. (a) Right eye. (b) Left eye. (a) After vitreous surgery and subretinal hematoma displacement, the retina was reattached. A spindle-like, hyperreflective lesion was observed. (b) The spindle-like, hyperreflective lesion could be seen under the neuroepithelial layer of the temporal side of the optic disc and macular lesion.

On the day following her initial visit, pars plana vitrectomy (PPV) using a 27-G three-port system (Constellation, Alcon, Fort Worth, Texas, United States) was performed on the right eye to treat the SMH secondary to CNV. We also performed simultaneous cataract surgery with implantation of an intraocular lens (IOLs). After core and peripheral vitrectomy, internal limiting membrane (ILM) peeling was conducted using Brilliant Blue G solution to reduce retinal traction. Recombinant tissue plasminogen activator (tPA, 50 *μ*g/0.4 mL) was injected into the subretinal space via a 38-G needle to liquefy the clot, targeting the area of greatest subretinal fluid without damaging the fovea. Finally, an air–fluid exchange was performed to displace the SMH away from the foveal center.Postoperatively, the patient was instructed to maintain a right lateral position to ensure effective displacement of the SMH. Two-week postsurgery, a single intravitreal injection of 0.5 mg/0.05 mL of ranibizumab (Lucentis, Novartis, Basel, Switzerland) was administered in the right eye to address the CNV and prevent further neovascularization. Intravitreal injection was deferred until 2 weeks after surgery to maintain intraocular gas fill and ensure appropriate imaging assessment postoperatively. However, 1 month following the initial surgery, a recurrence of the SMH was noted. To manage the recurrence, a second vitrectomy with tPA injection and air–fluid exchange was performed. Two weeks after the second surgery, an intravitreal injection of 2 mg/0.05 mL of aflibercept (Eylea, Bayer, Leverkusen, Germany) was administered in the right eye. An additional aflibercept injection was performed 1 month later to further manage CNV activity. Additionally, five intravitreal aflibercept injections were administered in the left eye over 6 months while monitoring for neovascular activity. Six years postoperatively, her BCVA improved to 20/125 in the right eye and remained stable at 20/20 in the left eye. The “peau d'orange” seen at the posterior pole of the fundus extends from the optic disc to the periphery in both eyes, with gray-white atrophic foci and pigmentation around the optic disc. The dark-brown AS spreads radially from the optic disc (Figure 4). Fundus examination revealed a reattached retina in the right eye with no active CNV, confirming the success of the surgical intervention and subsequent anti-VEGF therapy (Figures 5 and 6).

**Figure 7:**
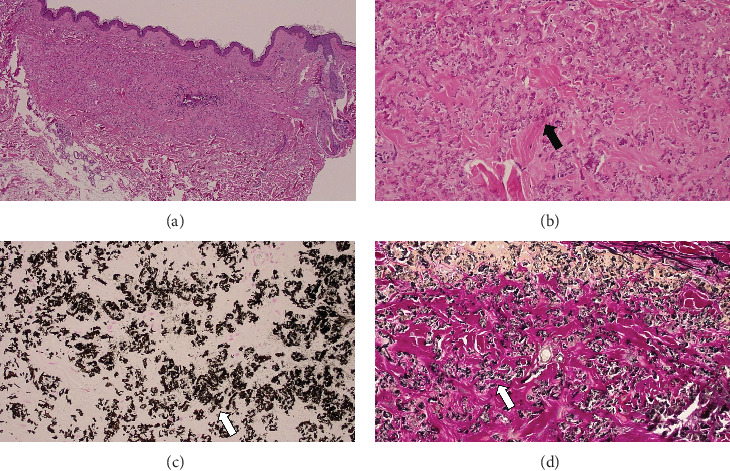
Pathological findings of skin lesions. (a) Objective x4, HE staining, fibrosis is seen in the dermis. (b) Objective x20, HE staining, large amounts of thread-like material (arrow) stained reddish purple are deposited. (c) Objective x20, Kossa staining, these materials are accompanied by calcification (arrow). (d) Objective x20, EVG staining, these are thought to be denatured elastic fibers (arrow).

These findings were consistent with the changes of the PXE fundus—AS. Due to her symptoms and the appearance of her neck skin resembling “chicken skin” (Figure 1), we recommended further examination at the Department of Dermatology, including tissue paraffin section and molecular pathology analyses (Figure 7), which confirmed the diagnosis of PXE.

## 3. Discussion and Conclusions

ASs are brittle lesions in BM often linked to systemic conditions with genetic components, such as PXE. More than half of AS patients appear to have an associated systemic condition [6, 7]. The prevalence of AS in PXE patients ranges from 59%–87% [7]. In comparison, the prevalence in Paget's disease is approximately 8%–15% [8], 0.9%–6% in sickle cell hemoglobinopathy [9, 10], and up to 30% in beta thalassemia patients over 50 years of age [11].

PXE is a genetic disorder characterized by abnormal elastin metabolism in elastic fibers, impacting organs with abundant elastic fibers, such as the skin, eyes, and cardiovascular system [1, 2]. Known also as the Grönblad–Strandberg syndrome when associated with retinal AS, PXE is the most common systemic disorder associated with AS. It occurs in about 1 in 100,000 to 200,000 people, typically between ages 20 and 30. PXE progresses slowly, with skin lesions that include symmetrical small yellow papules in intertriginous areas such as the axilla, lateral neck, inguinal region, and abdomen, which coalesce into reticulated or cobblestone plaques. Complications from abnormal elastin fibers in various organs can include cardiovascular abnormalities, gastrointestinal bleeding, epistaxis, and intracerebral hemorrhage. PXE is inherited in an autosomal recessive manner due to mutations in the ABCC6 gene [12], which encodes a protein functioning as a transmembrane transporter of small molecular weight conjugates [13]. The clinical features of PXE result from increased calcification in elastic fiber–rich connective tissues. It is hypothesized that the transporter may move molecules that inhibit calcification of elastic fibers, supported by mouse model data [14]. Various mutations cause dysfunction in this transporter, leading to the clinical phenotype of PXE.

BM, situated between the retinal pigment epithelium (RPE) and the choriocapillaris, facilitates nutrient and metabolite transport and acts as a barrier between the choroidal circulation and outer retinal layers. It contains significant elastin and collagen fibers, giving it unique biomechanical properties [7]. Mutations in the ABCC6 gene disrupt this metabolic pathway, causing calcium mineral accumulation in BM. A calcified BM becomes brittle and susceptible to crack-like lesions known as ASs [15]. The similar appearance of AS across different systemic disorders suggests a strong link to this metabolic pathway. Disruption of BM integrity compromises the retina–RPE barrier, potentially leading to CNV formation. While AS is not inherently hereditary, idiopathic cases with no apparent systemic disease also exist [16]. Though genetic testing is not mandatory for diagnosis, a multidisciplinary approach is essential for comprehensive patient care.

One of the most notable clinical features of AS in PXE patients is the “peau d'orange” appearance of the fundus, considered pathognomonic and typically the earliest fundus finding before AS development. Initially confined to the posterior pole, it may spread peripherally as the disease progresses. Fundus reflex changes vary with pigmentation, being more pronounced in darker-pigmented individuals. Peau d'orange may indicate interrupted calcification of BM [13]. AS can appear in various colors and be located around the optic disc or extend to the posterior pole, potentially affecting vision if encroaching on the fovea. Recurrent hemorrhages and choroidal ruptures are common, necessitating caution against strenuous activities. CNV formation in the macular region is a major complication, leading to significant vision loss. It can be classic or occult, with high incidence due to frequent AS occurrence in the posterior pole [7]. Polypoidal choroidal vasculopathy (PCV) and retinal telangiectasia have been reported in AS associated with PXE [17, 18]. Optic nerve head drusen are also known to be associated with PXE. In our case, a round, elevated lesion was observed at the inferior margin of the optic disc, raising the possibility of optic disc drusen. However, enhanced depth imaging OCT of the optic nerve was not performed at the time of examination, and thus, this finding could not be confirmed. We acknowledge this as a limitation of our case report and recommend OCT evaluation in similar future cases. This raises the possibility that in cases like ours, where there is a marked difference in presentation between the two eyes, PCV could coexist with PXE, contributing to the observed clinical asymmetry. Given that our patient was unable to undergo ICG angiography due to an allergy, a key diagnostic modality to differentiate between PCV and CNV was unavailable, leaving room for ambiguity in our diagnosis. Patients with AS, particularly those with PXE, are at risk for CNV and PCV, which can cause extensive subretinal hemorrhage. Another characteristic finding in PXE-associated AS is the presence of comet lesions, subretinal nodular lesions with a tapering tail, occurring more peripherally than peau d'orange [19]. These lesions are also seen in heterozygous carriers of ABCC6 mutations. Prompt recognition and treatment with anti-VEGF agents are crucial as CNV recurrence is common and affects visual outcomes.

Reports exist of patients undergoing vitrectomy for AS associated with proliferative diabetic retinopathy (PDR) [20] and those finding AS near a scleral buckle after retinal detachment surgery [21]. Still, there are no reports of vitreous surgery for extensive subretinal hemorrhage caused by AS. Jeong et al. reported that anti-VEGF alone has advantages for small SMH, with no significant difference in BCVA between PPV or pneumatic displacement (PD), and an advantage in BCVA for PPV + tPA for larger SMH [22]. Comparing visual outcomes and postoperative complications of two major treatments for SMH—PPV with tPA injection and PD with intravitreal tPA injection—showed that PPV with tPA offers a clear advantage. This method trends towards better visual acuity and is a significant predictor of improved visual acuity for small and medium SMH [23].

In summary, early detection of AS based on fundus findings is crucial. Thorough evaluation for concomitant systemic diseases, careful assessment of CNV activity, control of CNV using intravitreal injections of anti-VEGF drugs, and prompt vitreous surgery with tPA for SMH are essential steps in managing AS in PXE patients.

## Data Availability

The data that support the findings of this study are available from the corresponding author upon reasonable request.

## Nomenclature

OCT: optical coherence tomography
AS: angioid streaks
BCVA: best corrected visual acuity
BM: Bruch's membrane
CNV: choroidal neovascularization
ICG: indocyanine green
ILM: internal limiting membrane
PCV: polypoidal choroidal vasculopathy
PD: pneumatic displacement
PDR: proliferative diabetic retinopathy
PPV: pars plana vitrectomy
PXE: pseudoxanthoma elasticum
RPE: retinal pigment epithelium
SMH: submacular hemorrhage
tPA: tissue plasminogen activator
VEGF: vascular endothelial growth factor

## References

[1] Terry S. F., Uitto J., Adam M. P., Feldman J., Mirzaa G. M., Pagon R. A., Wallace S. E., Amemiya A. (2025). Pseudoxanthoma Elasticum. *GeneReviews®*.

[2] Cui C., Zhou Z., Zhang Y., Sun D. (2021). A Case Report: Pseudoxanthoma Elasticum Diagnosed Based on Ocular Angioid Streaks and the Curative Effect of Conbercept Treatment. *BMC Ophthalmology*.

[3] Gliem M., Finger R. P., Fimmers R., Brinkmann C. K., Holz F. G., Charbel Issa P. (2013). Treatment of Choroidal Neovascularization Due to Angioid Streaks: A Comprehensive Review. *Retina*.

[4] Mimoun G., Ebran J. M., Grenet T., Donati A., Cohen S. Y., Ponthieux A. (2017). Ranibizumab for Choroidal Neovascularization Secondary to Pseudoxanthoma Elasticum: 4-Year Results From the PIXEL Study in France. *Graefe's Archive for Clinical and Experimental Ophthalmology*.

[5] Gliem M., Birtel J., Herrmann P. (2020). Aflibercept for Choroidal Neovascularizations Secondary to Pseudoxanthoma Elasticum: A Prospective Study. *Graefe's Archive for Clinical and Experimental Ophthalmology*.

[6] Tsokolas G., Tossounis C., Tyradellis S., Motta L., Panos G. D., Empeslidis T. (2024). Angioid Streaks Remain a Challenge in Diagnosis, Management, and Treatment. *Vision*.

[7] Chatziralli I., Saitakis G., Dimitriou E. (2019). Angioid STREAKS: A Comprehensive Review From Pathophysiology to Treatment. *Retina*.

[8] Dabbs T. R., Skjodt K. (1990). Prevalence of Angioid Streaks and Other Ocular Complications of Paget’s Disease of Bone. *British Journal of Ophthalmology*.

[9] Ketner S., Moradi I. E., Rosenbaum P. S. (2015). Angioid Streaks in Association With Sickle Thalassemia Trait. *JAMA Ophthalmology*.

[10] Aessopos A., Voskaridou E., Kavouklis E. (1994). Angioid Streaks in Sickle-Thalassemia. *American Journal of Ophthalmology*.

[11] Barteselli G., Dell'Arti L., Finger R. P. (2014). The Spectrum of Ocular Alterations in Patients With *β*-Thalassemia Syndromes Suggests a Pathology Similar to Pseudoxanthoma Elasticum. *Ophthalmology*.

[12] Bergen A. A., Plomp A. S., Schuurman E. J. (2000). Mutations in ABCC6 Cause Pseudoxanthoma Elasticum. *Nature Genetics*.

[13] Gliem M., Zaeytijd J. D., Finger R. P., Holz F. G., Leroy B. P., Issa P. C. (2013). An Update on the Ocular Phenotype in Patients With Pseudoxanthoma Elasticum. *Frontiers in Genetics*.

[14] Jiang Q., Oldenburg R., Otsuru S., Grand-Pierre A. E., Horwitz E. M., Uitto J. (2010). Parabiotic Heterogenetic Pairing of *Abcc 6^−/−^*/*Rag1^−/−^* Mice and Their Wild-Type Counterparts Halts Ectopic Mineralization in a Murine Model of Pseudoxanthoma Elasticum. *American Journal of Pathology*.

[15] Booij J. C., Baas D. C., Beisekeeva J., Gorgels T. G. M. F., Bergen A. A. B. (2010). The Dynamic Nature of Bruch’s Membrane. *Progress in Retinal and Eye Research*.

[16] Gliem M., Wieg I., Birtel J. (2020). Retinal Findings in Carriers of Monoallelic ABCC6 Mutations. *British Journal of Ophthalmology*.

[17] Wong J. G., Qian K. Y. (2017). Long-Term Follow-Up of Polypoidal Choroidal Vasculopathy Secondary to Angioid Streaks Treated by Intravitreal Aflibercept and Ranibizumab. *Case Reports in Ophthalmology*.

[18] Nakagawa S., Yamashiro K., Tsujikawa A. (2013). The Time Course Changes of Choroidal Neovascularization in Angioid Streaks. *Retina*.

[19] Gass J. D. M. (2003). “Comet” Lesion: An Ocular Sign of Pseudoxanthoma Elasticum. *Retina*.

[20] Kakurai K., Hayashi M., Yamada K. (2017). A Case of Pseudoxanthoma Elasticum With Proliferative Diabetic Retinopathy. *BMC Ophthalmology*.

[21] Lee K. E., Thuma T. B. T., Salabati M., Sivalingam M. D., Pulido J. S., Gunton K. B. (2024). Pseudoxanthoma Elasticum-Associated Angioid Streaks Near a Scleral Buckle. *American Journal of Ophthalmology Case Reports*.

[22] Jeong S., Park D. G., Sagong M. (2020). Management of a Submacular Hemorrhage Secondary to Age-Related Macular Degeneration: A Comparison of Three Treatment Modalities. *Journal of Clinical Medicine*.

[23] Barzelay A., Daniels A., Cohen G. Y., Barak A., Schwartz S., Katz G. (2024). Pneumatic Displacement With Intravitreal tPA Injection Versus Vitrectomy With Sub Retinal tPA Injection in Small and Medium Sub Macular Hemorrhages-A Multicenter Comparative Study. *BMC Ophthalmology*.

